# Elucidation of chromatin loops among regulatory regions using Hi-TrAC

**DOI:** 10.3389/freae.2026.1858923

**Published:** 2026-06-19

**Authors:** Yaqiang Cao, Qingsong Tang, Keji Zhao

**Affiliations:** Laboratory of Epigenome Biology, Systems Biology Center, Division of Intramural Research, National Heart, Lung, and Blood Institute, National Institutes of Health, Bethesda, MD, United States

**Keywords:** chromatin accessibility, chromatin looping, enhancer-promoter interaction, Hi-TrAC, three-dimensional chromatin interaction

## Abstract

Cellular development and differentiation are critically regulated by the spatiotemporal expression of genes within the genome, which are packaged into a highly organized chromatin structure. Genes in the human genome are often regulated by long-distance promoter–enhancer interactions. To better understand gene expression regulation, it is essential to identify specific interactions between distal *cis*-regulatory elements and their target promoters. To this end, we developed Hi-TrAC, a technique that generates high-resolution interaction maps among accessible chromatin regions without relying on *in vitro* proximity ligation, while maintaining low sequencing cost. In addition, Hi-TrAC provides a genome-wide landscape of chromatin accessibility. In this article, we present a robust detailed protocol for performing Hi-TrAC assay.

## 1 Introduction

The human genome comprises approximately 20,000 protein-coding genes, yet these genes account for less than 2% of the total genomic sequence (International Human Genome Sequencing Consortium; Consortium, 2012; Nurk et al., 2022). The remaining vast majority of the genome consists of noncoding regions, much of which remains poorly characterized and is often referred to as genomic “dark matter” or even “junk DNA” (Consortium, 2012; Blaxter, 2010). These non-protein-coding regions play essential roles in regulating gene expression and maintaining cellular identity (Consortium, 2012). A key challenge in modern genomics is to decipher the functional significance of this noncoding portion of the genome and understand how it contributes to the regulation of gene activity (Claussnitzer et al., 2020).

Gene expression in humans is controlled by an extensive network of regulatory elements, estimated to include over 0.9 million putative *cis*-regulatory regions (Consortium, 2012; Roadmap Epigenomics et al., 2015; Consortium et al., 2020; Moore et al., 2026). These elements operate in a highly coordinated spatiotemporal manner, ensuring that genes are expressed at the right time, in the right cell type, and at appropriate levels. Among these regulatory elements, transcriptional enhancers are particularly important, as they can activate gene expression from considerable genomic distances. These regions are often identified experimentally as DNase I hypersensitive sites or, more broadly, as accessible chromatin regions, reflecting their open configuration and accessibility to transcription factors and regulatory proteins (Thurman et al., 2012; Buenrostro et al., 2013).

Understanding how these distal regulatory elements influence their target genes is critical for elucidating genome function. Unlike promoters, which are typically located immediately upstream of their target genes, enhancers can act over long genomic distances (Sanyal et al., 2012). This long-range regulation is mediated through the three-dimensional organization of chromatin, which brings enhancers and promoters into close spatial proximity despite their linear separation along the DNA sequence. Therefore, mapping the physical interactions between enhancers and promoters is a key step toward identifying functional regulatory relationships (Schoenfelder and Fraser, 2019).

To address the challenge of mapping long-range DNA contacts, chromosome conformation capture-based techniques such as Hi-C have been widely employed to investigate genome-wide chromatin interactions (Lieberman-Aiden et al., 2009; Rao et al., 2014; Dekker et al., 2026). These approaches have provided valuable insights into the three-dimensional architecture of the genome and revealed the presence of topologically associating domains (TADs) and larger-scale compartments (Lieberman-Aiden et al., 2009; Dixon et al., 2012; Nora et al., 2012). However, Hi-C and related methods often suffer from limited resolution and typically require extremely deep sequencing (several billions of reads) to detect specific interactions, particularly those involving distal regulatory elements (Rao et al., 2014; Hsieh et al., 2020). As a result, it remains difficult to pinpoint precise enhancer–promoter interactions using these techniques alone.

To overcome these limitations, alternative strategies–such as HiChIP (Mumbach et al., 2016), OCEAN-C (Li et al., 2018), HiCAR (Wei et al., 2022) and NicE-C (Luo et al., 2022) -- have been developed to capture regulatory chromatin interactions with higher specificity and efficiency. Despite differences in their enrichment strategies, these methods all rely on DNA-ligase-mediated proximity ligation to join spatially proximal chromatin fragments after fixation and fragmentation. This ligation step can introduce experimental background through nonspecific intermolecular ligation: DNA ends from unrelated complexes can occasionally be ligated by chance when intermolecular collisions occur during sample processing. Such spurious ligation products generate read pairs that do not necessarily represent true *in vivo* chromatin contacts. In Hi-C, such background is often reflected by elevated inter-chromosomal contact fractions, as showed by Nagano et al. that conventional in-solution ligation produced higher levels of trans-chromosomal contacts than in-nucleus ligation and a substantial fraction of these contacts resulted from spurious ligation events (Nagano et al., 2015). To circumvent this issue, a fundamentally different approach was developed that leverages the activity of a hyperactive DNA transposase Tnp derived from transposon Tn5, an enzyme capable of inserting exogenous DNA sequences into the genome. By engineering a Tnp complex that carries two binding motifs connected by a DNA linker, it becomes possible to simultaneously insert these motifs into two separate genomic regions (illustrated in Figure 1a). This process effectively creates a covalent linkage between distal regions of accessible chromatin without relying on traditional proximity ligation (Lai et al., 2018), thereby avoiding the random intermolecular DNA contacts typically introduced by conventional Hi-C-based methods. Following insertion, the linked genomic fragments can be isolated and sequenced, enabling the generation of a genome-wide map of spatial proximity among accessible chromatin regions (Liu et al., 2022). Because these regions are enriched for regulatory elements such as enhancers and promoters, limited sequencing depth of the libraries is sufficient to provide information on the specific interaction between enhancers and promoters genome-wide. Additionally, Hi-TrAC data also provide information on genome-wide chromatin accessibility and Topological Associated Domain (TAD) structure (Liu et al., 2022; Cao et al., 2023). Hi-TrAC detected numerous enhancer-enhancer interactions, which are shown to contribute to transcription as demonstrated by deleting the underlying enhancer using CRISPR (Liu et al., 2022). By integrating Hi-TrAC loop maps with transcription factor binding sites from ChIP-seq data and Hi-TrAC data generated after transcription factor knockdown, our previous study assigned different classes of chromatin loops to distinct regulatory factors. The analysis showed that CTCF and RAD21 preferentially contribute to longer-range, domain boundary-associated chromatin loops, whereas HCFC1 and ZNF143 preferentially contribute to shorter promoter- and enhancer-associated loops, revealing a division of labor model for different transcription factors in shaping the three-dimensional structure of the genome (Liu et al., 2022). Application of Hi-TrAC to mice T cell development revealed key regulatory elements required for the expression of signature cytokine genes such as *Ifng* and *Il4* (Cui et al., 2023; Liu et al., 2024).

**FIGURE 1:**
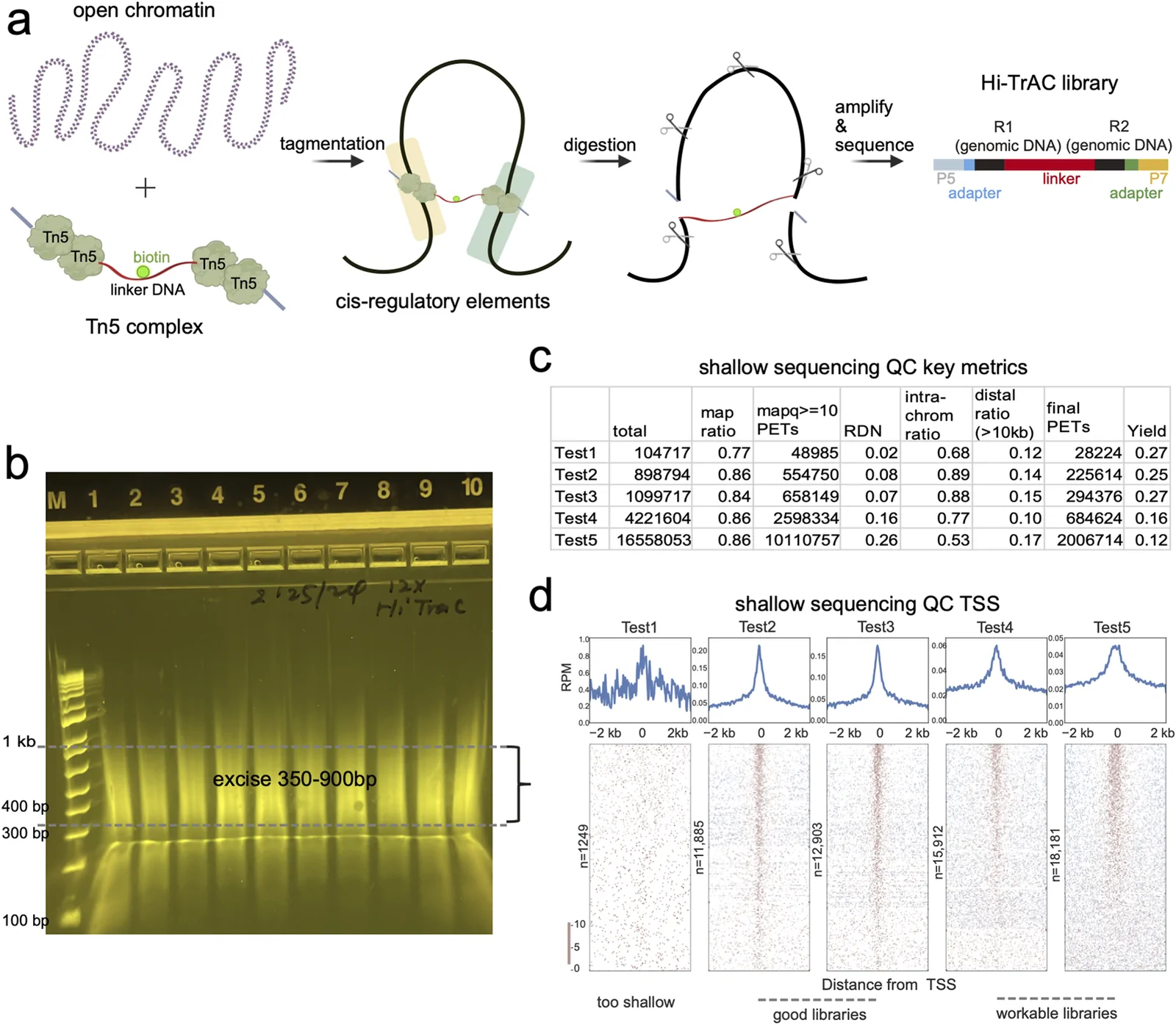
Hi-TrAC protocol and quality control metrics. **(a)** Hi-TrAC protocol flow chart. Hi-TrAC employs a complex formed between a bivalent linker and Tn5 to simultaneously insert two short oligonucleotide linkers into the genome and covalently connect two spatially-proximal DNA segments within open chromatin. Following this one-step integration, the resulting DNA-linker fragments are isolated and amplified for paired-end sequencing. This final sequencing step reveals the genomic locations of the co-captured DNA segments, allowing for the mapping of long-range chromatin interactions. **(b)** DNA fragment size selection by gel electrophoresis. This is a typical gel image from the protocol showing the amplified Hi-TrAC library (step 16). Fragments in the 350-900 bp range are excised for subsequent sequencing. **(c)** Table of key quality control (QC) metrics from shallow sequencing of five test libraries. This table presents key metrics from the shallow sequencing of five test libraries, obtained by processing the FASTQ files with tracPre2.py script from cLoops2 package. These metrics are crucial for assessing library quality and include the mapping ratio, intra-chromosomal PETs ratio, the number of final paired-end tags (PETs), and the proportion of long-distance (>10 kb) interactions (>0.1). RND stands for reads redundancy and indicates the library complexity. Yield is the ratio of final PETs to total PETs, used to estimate the required sequencing depth. **(d)** Transcription start site (TSS) enrichment plots. These plots offer a visual assessment of library quality signal-to-noise level based on shallow sequencing. The TSS signals at the center should be more than two-fold higher than the signals in the flanking regions.

Ultimately, Hi-TrAC offers a powerful means to directly probe the regulatory architecture of the genome and to better understand how noncoding elements orchestrate gene expression programs in human and mice cells.

## 2 Materials

### 2.1 Reagents

- Ultrapure water (KD medical cat# RGF-3410)
- 1M Tris-HCl, pH 7.4 (KD medical cat# RGF-3340)
- 1M Tris-HCl, pH 8.0 (KD medical cat# RGF-3360)
- Culture medium RPMI (Invitrogen, cat. no. 61870-036)
- FBS (Sigma-Aldrich, cat. no. F4135-500ML)
- Penicillin-Streptomycin (10,000 U/mL) (Gibco cat# 15140-122)
- Pierce methanol free 16% (wt/vol) formaldehyde (Thermo Fisher Scientific, cat. no. 28906)
- Glycine (Sigma-Aldrich, cat. no. 50046-250G)
- 1× PBS (Corning cat# 21-040-CV)
- 10% Tween 20 (Sigma-Aldrich, cat. no. 11332465001)
- Triton X-100 (Quality Biological, cat. no. A611-M143-13)
- 0.5 M EDTA (pH 8.0; KD Medical, cat. no. RGC-3130)
- 5 M sodium chloride (Quality Biological, cat. no. 351-036-721EA)
- 3 M sodium acetate (pH 5.2; Quality Biological, cat. no. 351-035-721EA)
- 10% (vol/vol) SDS (Quality Biological, cat. no. 351-032-101)
- Glycogen (20 μg/μL, Invitrogen, cat. no. 10814010)
- Proteinase K (recombinant, PCR grade; Sigma-Aldrich, cat. no. 3115836001)
- Ethanol (99.5% (vol/vol); Warner-Graham, cat. no. 64-17-5)
- dNTP Mix (10 mM ea; Thermo Fisher Scientific, cat. no. 18427013)
- NEB buffer r2.1 (New England BioLabs, cat. no. B6002S)
- rCutsmart Buffer (New England BioLabs, cat. no. B6004S)
- MluCI (New England BioLabs, cat. no. R0538L)
- NlaIII (New England BioLabs, cat. no. R0125L)
- T4 DNA Polymerase (New England BioLabs, cat. no. M0203L)
- T7 DNA ligase (New England BioLabs, cat. no. M0318L)
- StickTogether™ DNA Ligase Buffer(New England BioLabs, cat. no. B0535S)
- Phusion High-Fidelity PCR Master Mix with HF Buffer (New England BioLabs, cat. no. M0531S)
- MinElute Reaction Cleanup Kit (Qiagen, cat. no. 28206)
- Elution buffer (EB buffer) (Qiagen, cat. no. 19086)
- E-Gel 1 kb Plus DNA Ladder (Thermo Fisher Scientific, cat. no. 10488090)
- MinElute Gel Extraction Kit (Qiagen, cat. no. 28604)
- Qubit dsDNA HS Assay Kit (Thermo Fisher Scientific, cat. no. Q32854)
- RNase A (10 mg/mL, Thermo Fisher cat# 12091021)
- Phenol/chloroform/isoamyl alcohol, pH 6.7/8.0, VWR, 0883-100 mL)
- HisTnp [5 mg/mL or 90 μM; Tn5 transposase, home-made as described (Lai et al., 2018)]
- Ampure XP beads (Beckman Coulter cat# A63881)
- Streptavidin M280 beads (Thermo Fisher cat# 11205D)
- E-Gel EX Agarose Gels (2%; Thermo Fisher Scientific, cat. no. G401002)
- Illumina Multiplexing PCR i5 and i7 Index Primers Hi-TrAC adapter primers are all ordered from IDTDNA at 100 μM in IDTE (listed in Tables 1–3)

**TABLE 1:** i5 Index Primers.

i5-01	AAT​GAT​ACG​GCG​ACC​ACC​GAG​ATC​TAC​ACG​CTT​AGC​GAC​ACT​CTT​TCC​CTA​CAC​GAC​GCT
i5-02	AAT​GAT​ACG​GCG​ACC​ACC​GAG​ATC​TAC​ACT​ACG​AGC​CAC​ACT​CTT​TCC​CTA​CAC​GAC​GCT
i5-03	AAT​GAT​ACG​GCG​ACC​ACC​GAG​ATC​TAC​ACC​TTA​CCA​TAC​ACT​CTT​TCC​CTA​CAC​GAC​GCT
i5-04	AAT​GAT​ACG​GCG​ACC​ACC​GAG​ATC​TAC​ACG​GGT​TTT​GAC​ACT​CTT​TCC​CTA​CAC​GAC​GCT
i5-05	AAT​GAT​ACG​GCG​ACC​ACC​GAG​ATC​TAC​ACA​ATC​GCG​GAC​ACT​CTT​TCC​CTA​CAC​GAC​GCT
i5-06	AAT​GAT​ACG​GCG​ACC​ACC​GAG​ATC​TAC​ACC​ACG​TAT​CAC​ACT​CTT​TCC​CTA​CAC​GAC​GCT

**TABLE 2:** i7 Index Primers, * stands for a phosphorothioate bond.

M1	CAA​GCA​GAA​GAC​GGC​ATA​CGA​GAT​CGT​GAT​GTG​ACT​GGA​GTT​CAG​ACG​TGT​GCT​CTT​CCG​ATC*T
M2	CAA​GCA​GAA​GAC​GGC​ATA​CGA​GAT​ACA​TCG​GTG​ACT​GGA​GTT​CAG​ACG​TGT​GCT​CTT​CCG​ATC*T
M3	CAA​GCA​GAA​GAC​GGC​ATA​CGA​GAT​GCC​TAA​GTG​ACT​GGA​GTT​CAG​ACG​TGT​GCT​CTT​CCG​ATC*T
M4	CAA​GCA​GAA​GAC​GGC​ATA​CGA​GAT​TGG​TCA​GTG​ACT​GGA​GTT​CAG​ACG​TGT​GCT​CTT​CCG​ATC*T
M5	CAA​GCA​GAA​GAC​GGC​ATA​CGA​GAT​CAC​TGT​GTG​ACT​GGA​GTT​CAG​ACG​TGT​GCT​CTT​CCG​ATC*T
M6	CAA​GCA​GAA​GAC​GGC​ATA​CGA​GAT​ATT​GGC​GTG​ACT​GGA​GTT​CAG​ACG​TGT​GCT​CTT​CCG​ATC*T
M7	CAA​GCA​GAA​GAC​GGC​ATA​CGA​GAT​GAT​CTG​GTG​ACT​GGA​GTT​CAG​ACG​TGT​GCT​CTT​CCG​ATC*T
M8	CAA​GCA​GAA​GAC​GGC​ATA​CGA​GAT​TCA​AGT​GTG​ACT​GGA​GTT​CAG​ACG​TGT​GCT​CTT​CCG​ATC*T
M9	CAA​GCA​GAA​GAC​GGC​ATA​CGA​GAT​CTG​ATC​GTG​ACT​GGA​GTT​CAG​ACG​TGT​GCT​CTT​CCG​ATC*T
M10	CAA​GCA​GAA​GAC​GGC​ATA​CGA​GAT​AAG​CTA​GTG​ACT​GGA​GTT​CAG​ACG​TGT​GCT​CTT​CCG​ATC*T
M11	CAA​GCA​GAA​GAC​GGC​ATA​CGA​GAT​GTA​GCC​GTG​ACT​GGA​GTT​CAG​ACG​TGT​GCT​CTT​CCG​ATC*T
M12	CAA​GCA​GAA​GAC​GGC​ATA​CGA​GAT​TAC​AAG​GTG​ACT​GGA​GTT​CAG​ACG​TGT​GCT​CTT​CCG​ATC*T

**TABLE 3:** Hi-TrAC adapter primers.

Hi-TrAC_linker_top	/5Phos/CTGTCTCTTATACACATCTATACAGAGGAC/iBiodT/CCGGAGCAACCACAGACAAGATGTGTATAAGAGACAG
Hi-TrAC_linker_bottom	/5Phos/CTGTCTCTTATACACATCTTGTCTGTGGTTGCTCCGGAGTCCTCTGTATAGATGTGTATAAGAGACAG
Hi-TrAC_short_adapter_top	TCC​CTC​GCG​CCA​TCA​GAG​ATG​TGT​ATA​AGA​GAC​AG
pMENTS	/5Phos/CTGTCTCTTATACACATCT
NlaIII-adapter-top	/5Phos/A GAT CGG AAG AGC ACA CGT CT
NlaIII-adapter-btm	ACA​CTC​TTT​CCC​TAC​ACG​ACG​CTC​TTC​CGA​TCT​CAT​G
MluCI-adpt-top	/5Phos/AATTA GAT CGG AAG AGC ACA CGT CT
MluCI-adpt-bottom	ACA​CTC​TTT​CCC​TAC​ACG​ACG​CTC​TTC​CGA​TCT

### 2.2 Equipment

- Magnetic rack (Invitrogen cat# 12321D)
- DiaMag 0.2mL - magnetic rack (Diagenode cat# B04000001)
- Labquake shaker rotisserie (rotator, Thermo Scientific, model # 400110. We put this rotator in a 37 °C incubator)
- SafeSeal Microcentrifuge tubes (1.7 mL, BioScience, Inc. cat#11170)
- SafeSeal Microcentrifuge tubes (0.65 mL, BioScience, Inc. cat#11130)
- 0.2 mL 8-strip tubes (Thomas Scientific cat# 1148A16)
- Tabletop microcentrifuge (model no. MicroStar 17R; VWR, cat. no. 521-1647)
- Precision general-purpose water baths (Thermo Fisher Scientific, cat. no. 2824)
- Thermal cycler (model no. PTC-200; MJ Research, cat. no. 8252-30-0001)
- E-Gel Power Snap Electrophoresis System (Thermo Fisher Scientific, cat. no. G8300)
- Qubit fluorometer (Life Technologies, cat. no. Q32866)

### 2.3 Preparation of buffers and oligonucleotide adaptors

- Hi-TrAC Lysis buffer:
  50 mM Tris-HCl (pH 7.4), 10 mM NaCl, 1 mM EDTA, 0.1% Triton X-100, store at 4 °C.
- 10x Tnp reaction buffer:
  0.5.M Tris-acetate (pH 7.5), 1.5 M potassium acetate, 100 mM magnesium acetate, 40 mM spermidine. Adjust pH to 7.5 with KOH or HAc. Aliquot and store at −20 °C.
- HisTnp elution buffer:
  50 mM Tris-HCl pH 8.0, 1 M NaCl, 250 mM imidazole, 0.1% Triton X-100. Store at 4 °C.
- Elution/50% glycerol:
  Mix equal volumes of HisTnp elution buffer and glycerol. Store at −20 °C.
- 1x B&W&T buffer:
  5 mM Tris-HCl pH 7.4, 1 M NaCl, 0.5 mM EDTA, 0.05% Tween-20.
- Anneal adapters:
  1. 50 μM bivalent linker1042024: mix equimolar amount of oligo Hi-TrAC_linker_top and oligo Hi-TrAC_linker_bottom;
  2. 50 μM short adapter1042024: mix equimolar amount of oligo Hi-TrAC_short_adapter_top and oligo pMENTS;
  3. 50 μM NlaIII-adapter: mix equimolar amount of oligo NlaIII-adapter-top and oligo NlaIII-adapter-btm;
  4. 50 μM MluCI-adapter: mix equimolar amount of oligo MluCI-adpt-top and oligo MluCI-adpt-bottom.

Once the oligos are mixed as described above, put them in a thermocycler, heat to 95 °C for 5 min, then decrease temperature by 0.1 °C every 10 s for 700 cycles, hold at 25 °C for 10 min, then 4 °C. Aliquot and store at −20 °C.

## 3 Experimental procedures

### 3.1 Day 1: Tnp complex assembly and Hi-TrAC reaction (∼1 h then overnight)

#### 3.1.1 Assemble 330 μL Hi-TrAC complex as following

- Add 50 μL Elution Buffer (EB) into one 1.7 mL SafeSeal Microcentrifuge tube. Add 86 μL glycerol. Mix well.
- Add 24 μL 50uM short adapter1042024 and 12 μL 50uM bivalent linker1042024. Mix well.
- Add 126 μL Elution/50%glycerol. Mix well.
- Add 32 μL HisTnp (5 mg/mL or 90 μM) at last, mix thoroughly by pipetting. The ideal Hi-TrAC complex contains one bivalent linker, two short adapters, and four HisTnp molecules. For the standard 330 µL assembly, this corresponds to 0.6 nmol bivalent linker, 1.2 nmol short adapter, and ∼2.88 nmol HisTnp, giving a final assembled complex concentration of approximately 1.8 µM. If the HisTnp stock concentration differs, adjust the added volume to keep the same molar amount of HisTnp in the assembly reaction.
- Incubate at room temperature for 20 min. Store the complex at 4 °C for up to 6 months.

#### 3.1.2 Cell preparation

- GM12878 cells were cultured and fixed as described as before (Liu et al., 2022). Briefly the cells were cultured in complete culture medium (RPMI, 10%FBS and 100 U/mL Penicillin-Streptomycin). Cells were harvested and resuspended in the same media at 1 million/mL concentration at room temperature. Add 1% formaldehyde (final concentration) and gently rock for 10 min at room temperature. Add 1/10 volume of 1.25 M Glycine to quench for 5 min. Spin at 500x g at 4 °C for 5 min. Wash the cells pellet twice with ice-cold PBS. The fixed cell aliquots resuspended in PBS are stored in −80 °C freezer.
- Take out 1million fixed cells from −80 °C freezer and thaw at room temperature.
- Spin at 3,000 rpm at 4 °C for 2 min. Discard PBS supernatant.
- Resuspend in 200 μL Hi-TrAC Lysis buffer per 1 million cells. Incubate on ice 10 min.
- Spin at 3,000 rpm at 4 °C for 2 min. Discard supernatant.
- Resuspend in 100 μL 1x Tnp reaction buffer diluted from 10x Tnp reaction buffer. Transfer to 0.65 mL tube.

#### 3.1.3 Adapter insertion and chromatin tagmentation

- Add 1 μL assembled Hi-TrAC complex to cells. Mix by brief vertexing.
- Incubate with gentle rocking on a Labquake shaker rotisserie at 37 °C for 20 h or overnight.

### 3.2 Day 2: reverse cross-linking (0.5 h, then overnight)

#### 3.2.1 Stop reaction and reverse cross-linking

- Add 5 μL 0.5 M EDTA to the reaction mixture and mix well.
- Add 95 μL freshly prepared stop mix (6 μL 10% SDS, 84 μL 1x TE pH 8, 5 μL Proteinase K). The final volume is 200 μL. Vortex for 2 s, spin for 2 s.
- Incubate at 65 °C overnight.

### 3.3 Day 3: Hi-TrAC library prep steps (∼9 h)

#### 3.3.1 RNA removal (∼1 h)

- Add 2 μL RNase A (10 mg/mL) to the reaction mixture, incubate at 37 °C for 30 min.

#### 3.3.2 DNA extraction/precipitation (∼1 h)

- Transfer 200 μL Hi-TrAC reaction mix to new 1.7 mL SafeSeal Microcentrifuge tubes. If the volume is less than 200 μL, add EB buffer to make up the volume.
- Add 200 μL phenol/chloroform/isoamyl alcohol. Vortex 30 s, centrifuge at 16,000 x g at 4 °C for 3 min.
- Take out the bottom phase and discard, spin the tubes at 16,000 × g at 4 °C for 10 min. Transfer ∼200 μL clear upper phase to 21 μL mix (1 μL glycogen, 20 μL 3 M sodium acetate) in a new tube.
- Add 500 μL 100% ethanol, mix well by vortex briefly. Incubate on dry ice for 30 min.
- Stop: the samples can be stored at −80 °C freezer overnight and continue later.

#### 3.3.3 Ethanol wash

- Thaw the tubes, centrifuge at 16,000 × g at 4 °C for 10 min. Remove supernatant.
- Add 1 mL 80% ethanol to the pellet, centrifuge at 16,000 × g at 4 °C for 5 min. Repeat once. Remove as much ethanol as possible.

#### 3.3.4 Gap repair (∼1 h)

- Air-dry pellets at room temperature for 3 min. Resuspend pellets in 50 μL gap-repair mix containing 5 μL 10 × NEBuffer r2.1, 1 μL dNTP Solution Mix (10 mM each), 1 μL T4 DNA Polymerase, 43 μL ddH_2_O.
- Incubate at room temperature for 30 min.
- Add 10 μL 100 mM EDTA to stop reaction. Vortex, then spin for 30 s so that there is no liquid on the wall of the tube. This step is critical because, in the following step, after adding 36 μL beads, the beads will settle to the bottom of the tube instead of spreading to the entire inner wall during rotation.

#### 3.3.5 Ampure XP bead cleanup (∼1 h)

- Add 36 μL Ampure XP beads. Let beads settle for 1 min, followed by rocking gently at room temperature for 30 min. The liquid and beads will stay at the bottom of the tube.
- Place on magnet for 2 min, remove supernatant. Wash beads twice with 500 μL 80% ethanol.

#### 3.3.6 Restriction enzyme digestion (∼1 h)

- Air-dry beads 3 min. To each tube, add 100 μL digestion mix containing 10 μL 10x rCutSmart, 4 μL MluCI, 4 μL NlaIII, 82 μL ddH_2_O. Mix well by pipetting or brief vortexing.
- Incubate at 37 °C for 30 min. Put the tubes on a magnet for 2 min, collect supernatant.
- Wash beads with 50 μL 10 mM Tris-HCl pH 8.0, collect supernatant and combine with the supernatant collected above.

#### 3.3.7 Column cleanup

- Add 450 μL ERC buffer to the 150 μL pooled supernatant. Vortex briefly, spin down in a microfuge for 10 s. Load the 600 μL mix on MinElute column, centrifuge at 15,000rpm at room temperature for 2 min.
- Wash with ethanol: add 600 μL 70% ethanol (PE buffer) to the column, centrifuge at 15,000 rpm at room temperature for 1 min. Dump the flowthrough and spin again for 1 min.
- Elute the bound DNA: transfer the MinElute column onto a new 1.7 mL SafeSeal Microcentrifuge tube, add 50 μL warm (65 °C) EB to the column, incubate at room temperature for 2 min, centrifuge at 15,000rpm at room temperature for 1 min.

#### 3.3.8 Streptavidin bead binding (∼0.5 h)

- Prepare 20 μL Streptavidin M280 beads/sample: take out 20 μL Streptavidin M280 beads from the stock and wash 3x with 1 mL 1x B&W&T buffer.
- Resuspend the beads in 100 μL 1x B&W&T + 50 μL 2x B&W buffer per sample.
- Add 150 μL beads to 50 μL DNA collected above, rotate at room temperature for 30 min. Put the tubes on magnet for 2 min, remove supernatant.

#### 3.3.9 Bead washing (∼0.5 h)

- Wash twice with 1 mL 1x B&W&T buffer: add the buffer to the tube, invert 16x, briefly spin the tubes for 2 s, then put on magnet for 2 min to collect the beads before removing supernatant.
- Wash once with 1 mL 1x TBS pH 7.4: add the buffer to the tube and wash as above.

#### 3.3.10 Adapter ligation and beads washing (∼1 h)

- Prepare 95 μL adapter mix containing 50 μL 2x T7 DNA Ligase Buffer, 35 μL ddH_2_O, 5 μL 50 μM MluCI-adapter, 5 μL 50 μM NlaIII-adapter.
- Add 95 μL adapter mix to beads, vortex briefly to mix well. Add 5 μL T7 DNA ligase to the reaction mix and vortex gently to mix well. Rotate the tubes at room temperature for 30 min.
- Put the tube on magnet for 2 min to collect the beads, remove supernatant.
- Put the tubes on a rack and add 1 mL 1x B&W&T buffer. Close the cap. Invert the rack 16 times to wash the beads. Briefly spin the tubes for 2 s and put the tubes on a magnet for 2 min to collect the beads, remove supernatant. Repeat this beads washing 4 more times.
- Wash the beads once with 1 mL 1x TBS pH 7.4.

#### 3.3.11 PCR amplification (0.5 h)

- Prepare 40 μL PCR mix containing 18 μL ddH_2_O, 20 μL 2x Phusion HF Master Mix, 1 μL Illumina Multiplexing PCR i5 Index Primer such as i5-01 (10 μM), 1 μL Illumina Multiplexing PCR i7 Index Primer such as M1 (10 μM).
- Remove the TBS buffer from the beads while keeping the tube on magnet.
- Resuspend beads with 20 μL PCR mix, transfer to PCR tubes. Add the remaining 20 μL PCR mix to the original tube and transfer everything to the same PCR tube to combine the two 20 μL PCR mix.
- Run PCR: 98 °C 30 s, 12 cycles of (98 °C 10 s, 65 °C 30 s, 72 °C 30 s), 72 °C 5 min, hold 4 °C.
- **Stop:** the samples can be held at 4 °C overnight.

#### 3.3.12 Size selection by gel electrophoresis (∼0.5 h)

- Put the PCR strip on a DiaMag 0.2 mL magnetic rack to separate the soluble PCR products from the beads.
- Load 20 μL PCR product per lane on 2% E-gel, run 26 min. Excise 350–900 bp.
- Extract DNA of 350–900 bp using MinElute Gel Extraction Kit, elute with 14 μL EB.
- Note: Gel excision is recommended for Hi-TrAC library size selection. Although AMPure XP bead-based size selection can be convenient, we do not use it as the default method because PCR primer dimers and short nonspecific products are close to the lower end of the desired 350–900 bp library range. Gel excision provides more precise removal of these unwanted products.

#### 3.3.13 Library quantification (∼0.5 h)

- Measure concentration using Qubit dsDNA HS Assay Kit. (expect ∼1 ng/μL).

#### 3.3.14 Sequencing

- The libraries were sequenced in Illumina platforms in 50-8-8–50 PE format.

## 4 Data analysis

- The analysis of Hi-TrAC data is consistently performed using a comprehensive bioinformatic pipeline centered around the cLoops2 software suite (Cao et al., 2022), which was specifically designed for this type of chromatin interaction data. The process can be divided into two main stages: raw data processing and basic analysis, summarized as Figure 2a.
- Raw data processing: The initial raw data processing stage converts sequencing reads into a clean, analyzable format using the tracPre2.py script. The process begins by computationally removing the Hi-TrAC-specific bivalent linker sequence from the raw FASTQ reads. Subsequently, the trimmed reads are aligned to a reference genome using the Bowtie2 aligner, typically with high-sensitivity parameters. To ensure the highest data quality, a multi-step filtering protocol is applied: ambiguously mapped reads with a low mapping quality (MAPQ <10) are discarded, identical paired-end tags (PETs) arising from PCR amplification bias are removed. In addition, PETs whose two genomic ends map within 1 kb of each other but lack detectable full or partial Hi-TrAC bivalent-linker sequence in either read are classified as proximal no-linker PETs and removed as background reads. These PETs may include linker-containing products that are not recognized because 50 bp paired-end reads do not reach the linker sequence, but many likely represent local Tnp insertion or fragmentation products rather than bivalent-linker-mediated chromatin contacts. The final output of this pipeline is a high-quality BEDPE (Paired-End BED) file containing unique PETs, which along with a quality control report, serves as the primary input for all subsequent downstream analyses.
- Basic analysis: Following the initial processing of raw data, the cLoops2 software package is employed for the foundational analysis and identification of key chromatin features. The package utilizes a density-based clustering algorithm (blockDBSCAN) to directly analyze the genomic coordinates of paired-end tags (PETs). This approach allows for the concurrent calling of both peaks and chromatin loops. Peaks, which represent regions of open and accessible chromatin analogous to those found in ATAC-seq, are identified from short-range interactions (callPeaks). In parallel, chromatin loops, representing physical connections between distant genomic elements, are identified from long-range interactions (callLoops). Furthermore, cLoops2 can identify active, TAD-like structures, termed “active sub-TADs,” by calculating a novel segregation score (callDomains). For comparative studies, the pipeline supports the identification of differential loops between conditions (callDiffLoops). The resulting features can be visualized through contact maps and arc plots, as shown in a typical high-resolution map of Hi-TrAC data (Figure 2b), or used for other downstream applications like aggregate analysis and integration with other omics data to elucidate biological function.

**FIGURE 2:**
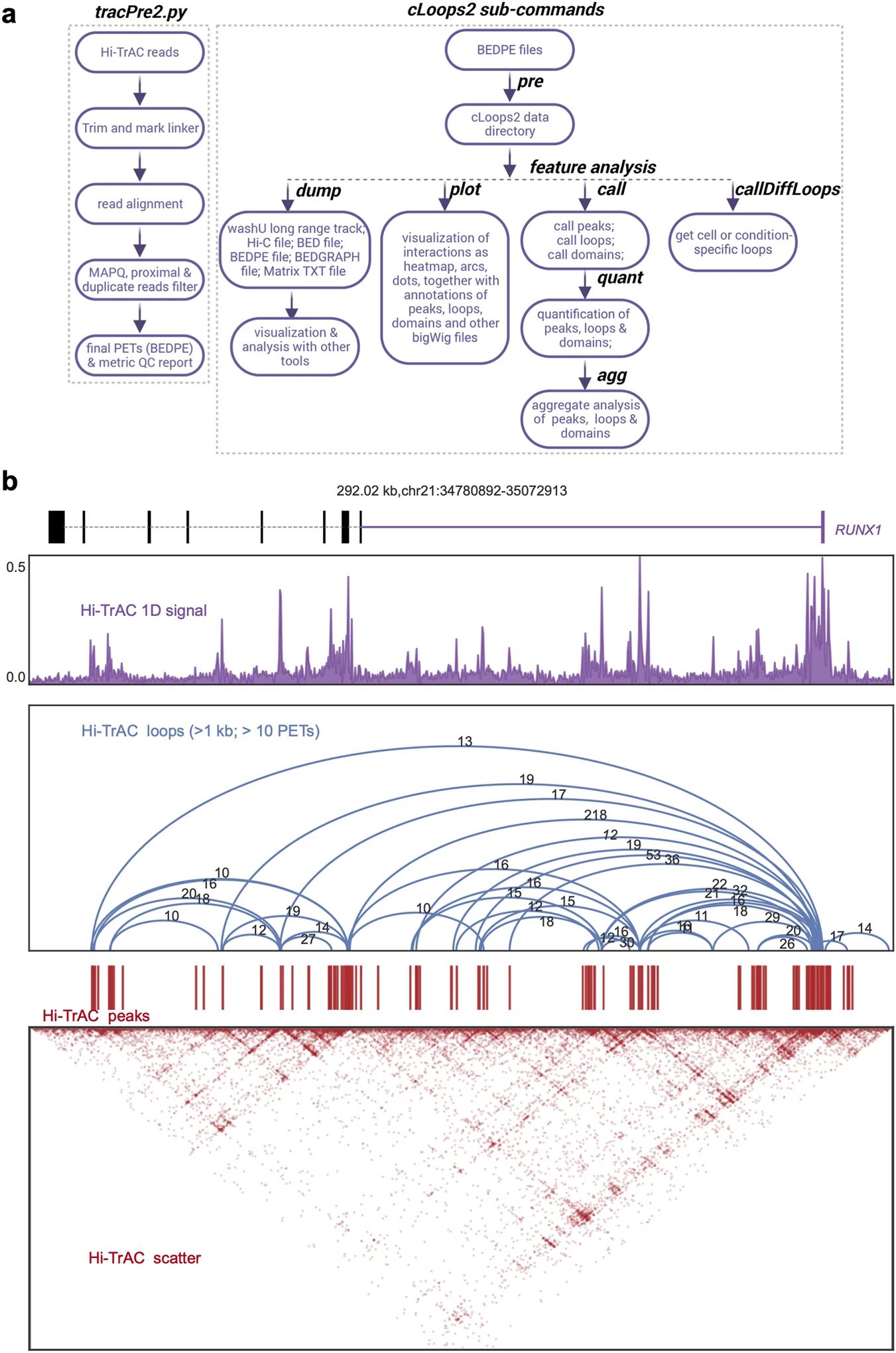
Bioinformatic analysis and visualization of Hi-TrAC data. **(a)** Basic Hi-TrAC data analysis workflow with cLoops2. This flowchart illustrates the bioinformatic pipeline for processing Hi-TrAC data. Initially, raw sequencing reads are processed with tracPre2.py for essential steps like linker trimming, alignment, and filtering of low-quality or duplicate reads. This produces a set of high-confidence paired-end tags (PETs) and a quality control report. Subsequent in-depth analysis is carried out using the cLoops2 suite of tools, which allows for downstream applications such as loop and domain calling, quantification, aggregate analysis, and visualization. **(b)** Visualization of chromatin interactions detected by Hi-TrAC at the *RUNX1* locus in GM12878 cells. This panel provides an example of Hi-TrAC data visualization for a 292 kb region encompassing the RUNX1 gene. Hi-TrAC data is presented in four ways from top to bottom: the one-dimensional Hi-TrAC signal track, called Hi-TrAC loops, called Hi-TrAC peaks, and the Hi-TrAC PET scatter/contact map. The one-dimensional Hi-TrAC signal track showing the linear genomic distribution of Hi-TrAC signal intensity across the locus, analogous to an ATAC-seq accessibility coverage profile. The arc diagram highlighting significant long-range chromatin loops (interactions >1 kb supported by >10 PETs). The ATAC-seq like peaks called directly from Hi-TrAC data. The scatter plot (contact map) where each dot represents a paired-end tag, providing a comprehensive view of all detected chromatin contacts within the region. Concordance among 1D signal, called Hi-TrAC peaks, loop anchors, and focal PET clusters indicates high-quality Hi-TrAC data and supports the identification of regulatory chromatin interactions. The Hi-TrAC data and loops were obtained from our previous study (Liu et al., 2022).

## 5 Anticipated results

- After PCR amplification, the PCR products should show up as a smear of 300 to 1,000 bp on gel as in Figure 1b. After shallow sequencing (about 1–5 million reads per library), the resulting library should meet key quality metrics: the proportion of long-distance paired-end tags (PETs) should exceed 10%, the intra-chromosomal PETs ratio should be greater than 50% (Figure 1c), and the signal enrichment around transcription start sites (TSSs) should be at least two-fold higher than the background (Figure 1d).
- Upon deep sequencing and data processing (50–100 million finally PETs), a high-quality library will reveal both accessible chromatin features and dense long-range interaction regions. As illustrated at the RUNX1 locus from human GM12878 cells in Figure 2b, the one-dimensional Hi-TrAC signal track shows the linear distribution of Hi-TrAC signal intensity across the locus, analogous to an ATAC-seq-like accessibility profile. Hi-TrAC peaks called from the same dataset mark accessible regulatory regions. The arc plot displays called chromatin loops greater than 1 kb and supported by more than 10 PETs, with the number above each arc indicating the supporting PET count. The scatter/contact map shows individual PETs across the locus; focal off-diagonal PET clusters correspond to long-range chromatin interactions. In high-quality data, called Hi-TrAC peaks, loop anchors, and focal PET clusters should show concordant patterns, supporting the identification of regulatory chromatin interactions. In contrast, weak peak enrichment, diffuse off-diagonal signal, or lack of focal loop structures may indicate insufficient sequencing depth, low library complexity, or high background.

## 6 Troubleshooting

- Low yield after PCR in step 16: if the PCR product smear is faint, put the rest 20 ul PCR back to a thermocycler for 5 more cycles amplification.
- Too low PCR yield may be due to poor tagmentation: Check Tnp tagmentation activity for each batch of Tn5 preparation.
- Measuring Tnp activity: Tnp activity should be validated for each new Tnp preparation. A simplified *in vitro* assay can be performed (Lai et al., 2018). First, assemble a test transposome using only the short adapter and the Tnp preparation, maintaining the same molar ratio between Tnp and ME ends as used in the complete Hi-TrAC complex. Add the assembled transposome to a tagmentation reaction containing 50–100 ng plasmid DNA or high-molecular-weight DNA in 1× Tn5 reaction buffer, and incubate at 37 °C for 30 min. Purify the tagmented DNA using a MinElute column and analyze the size distribution on a 1–2% agarose gel or 2% E-Gel. Successful Tn5 activity is indicated by conversion of the input DNA into a broad DNA smear. A no-Tnp reaction should be included as a negative control, and, when possible, a previously validated Tnp batch should be included as a positive control.
- High background: High-background libraries often show low TSS enrichment, a low distal interacting PET ratio, and a high inter-chromosomal PET ratio. In shallow-sequencing quality control, a good library should generally show at least two-fold signal enrichment around TSSs compared with flanking background regions, a distal PET ratio greater than 10%, and an intra-chromosomal PET ratio greater than 50%. Libraries failing these criteria often also show a flat or pervasive one-dimensional accessibility-like signal and diffuse PET signal in contact maps without clear focal loop enrichment.
- To reduce background: Ensure complete removal of free adapters and genomic DNA fragments without biotin-adapter insert in step 14 by 5 times wash. To avoid carryover, transfer the beads/buffer to a new 1.7 mL SafeSeal Microcentrifuge tube after 5th wash.
- The fraction of proximal no-linker PETs may increase when the ratio of Hi-TrAC complex to cell number is not optimal, which varies depending on cell types. To reduce this kind of background, we recommend titrating the Hi-TrAC complex-to-cell ratio when new cell types are used for the assay.
- The library complexity requires sufficient number of cells used for the assay. The minimal cell number required for the assay varies depending on the cell type: while 10–100 K cells may be sufficient for mouse embryonic stems, it may require 1 million T cells to generate a high-quality library.

## 7 Limitations

Although Hi-TrAC avoids conventional proximity ligation, several limitations should be considered. Because Hi-TrAC relies on Tn5-mediated insertion, the assay is influenced by chromatin accessibility and Tn5 insertion preference and therefore preferentially detects interactions involving open chromatin regions. The subsequent MluCI/NlaIII digestion and size-selection steps may also introduce recovery bias related to restriction-site distribution, incomplete or excessive digestion, and fragment size. In addition, the detection limit depends on library complexity, sequencing depth, and interaction frequency; shallow sequencing is useful for quality control, whereas robust loop detection generally requires 50–100 million final PETs. The current protocol was optimized using 1 million fixed cells per reaction, and lower input amounts require further optimization. Data analysis can also be affected by mappability, duplicate removal, linker detection, filtering thresholds, and loop-calling parameters. Finally, although Hi-TrAC provides an ATAC-seq-like accessibility signal, it is not suitable for transcription-factor footprinting based on precise Tn5 insertion patterns. Because the current protocol involves extended tagmentation followed by restriction digestion and adapter ligation, the final Hi-TrAC reads are located near, but do not necessarily mark, the exact original Tn5 insertion sites. Therefore, Hi-TrAC interactions should be interpreted as spatial proximity among accessible chromatin regions, and functional relevance or transcription-factor occupancy should be validated with orthogonal assays when needed.

## Data Availability

Publicly available datasets were analyzed in this study. This data can be found here: GEO with accession of GSE180175.
